# Bladder exstrophy on YouTube: an evaluation of quality, actionability, and user engagement

**DOI:** 10.1007/s00345-026-06229-z

**Published:** 2026-04-11

**Authors:** Aidan S. Weitzner, Alex Simpkins, Jason Yang, Tanisha Martheswaran, Madilynn Perrigo, Chad B. Crigger

**Affiliations:** James Buchanan Brady Urological Institute, Johns Hopkins University School of Medicine, 200 North Wolfe Street, Baltimore, MD 21287 USA

**Keywords:** Pediatric urology, Bladder exstrophy, Social media

## Abstract

**Purpose:**

To assess the quality and engagement of bladder exstrophy content on YouTube using validated assessment tools, with the goal of identifying gaps in accessible, high-quality online education. These findings also have broader implications for the interplay of online information and rare conditions.

**Methods:**

We reviewed the first 300 YouTube videos relevant to the term “bladder exstrophy” published prior to March 2025. Video quality was assessed using the Patient Education Materials Assessment Tool (PEMAT) and JAMA benchmark criteria. Engagement (views, likes, comments), presenter credentials, and targeted audience were extracted. Spearman correlation coefficients were used to examine the relationship between viewer engagement and quality scores.

**Results:**

133 videos met inclusion criteria. Academic institutions produced 60% of videos. The median video length was 5 min with a median view count of 204. Patient-created videos had the highest Video Power Index (VPI), however this was not statistically significant (*p* = 0.11). Median understandability (PEMAT-V) was high (89), while actionability (PEMAT-A) was low (0). Academic videos had significantly higher PEMAT-V (*p* = 0.002) and JAMA benchmark scores (*p* < 0.001) compared to patient, layperson content. No significant correlations were found between video quality scores and user engagement metrics.

**Conclusion:**

Content on bladder exstrophy garners limited viewership and engagement but remains an important educational resource. Videos produced by academic institutions demonstrated the highest quality scores, however, lacked actionable guidance. Higher quality was not associated with increased engagement, highlighting a critical gap in the availability of educational, user-friendly videos that support both understanding and anticipatory guidance for the management of bladder exstrophy.

**Supplementary Information:**

The online version contains supplementary material available at 10.1007/s00345-026-06229-z.

## Introduction

 Bladder exstrophy is a rare and complex congenital anomaly encompassing a spectrum of midline defects involving the urinary bladder, abdominal wall, pelvic bones, and external genitalia [1]. Bladder exstrophy affects an estimated 2.1 to 5.2 per 100,000 live births, with a higher prevalence among males [2, 3]. The etiology is likely multifactorial, involving both genetic and environmental contributors [4, 5]. Given its anatomical and clinical intricacies, bladder exstrophy poses significant challenges in diagnosis, surgical reconstruction, and long-term care. Although advances in surgical and multidisciplinary care have markedly improved outcomes, affected individuals often face lifelong issues related to urinary incontinence, sexual function, body image, and psychosocial well-being [6–8]. 

Amid the complexities of this diagnosis, many families turn to the internet and social media, which have rapidly transformed health information dissemination. Platforms like YouTube, Twitter, and TikTok have become key sources of medical content for patients and caregivers [9, 10]. While these platforms can enhance health literacy and reduce stigma, they also carry risks related to misinformation, bias, and poor content quality [11]. Prior studies in urology have shown that much of the information available on social media is of moderate to poor quality, often influenced by commercial interests [12–14]. This underscores the need for critical evaluation of online health resources, particularly for multifaceted conditions like bladder exstrophy.

Despite growing reliance on social media, the quality of bladder exstrophy-related content on YouTube remains unexamined. Research on more common urologic conditions has shown that high engagement does not indicate high-quality or accurate information [15, 16]. As specialized knowledge is essential to understanding bladder exstrophy, misinformation and lack of actionable content may negatively affect patient care. To date, no study has systemically evaluated the quality of YouTube content on bladder exstrophy. This study aims to assess the quality and engagement of bladder exstrophy videos using validated tools and to explore differences based on authorship, intended audience, and region.

## Methods

### Video selection

On February 27, 2025, we conducted an automated YouTube (www.youtube.com) search for the term “bladder exstrophy” using a Python script to minimize algorithmic bias and personalization (Supplement 1) [17]. The first 300 videos returned by the platform’s default relevance-based algorithm were screened. Metadata including video title, upload date, author, and number of likes and views were extracted using the YouTube Search API in Python (Version 3.10.12; Python Software Foundation). Videos were independently reviewed by two authors (AW, AS, JL), with discrepancies resolved by a third reviewer. Inclusion criteria were: (1) relevant to bladder exstrophy, (2) English language, (3) inclusion of audio or text, (4) duration < 30 min and not part of a lecture series or conference, and (5) not procedural in nature. Videos greater than thirty minutes and conference lectures were excluded to focus on patient-oriented content. Procedural videos were excluded as they target surgical trainees instead of a lay/patient audience.

### Video engagement characteristics

For each eligible video, we recorded the author type (academic, patient, or layperson), intended audience (patients, healthcare professionals, or general public), and geographic origin. Video popularity was assessed by view count. To quantify user engagement, we calculated the video power index (VPI) as:

$$\:VPI=\left(\frac{Likes}{Views}\right)*100$$

### Quality, understandability, and actionability outcomes

Each video was evaluated using two validated instruments. The Patient Education Materials Assessment Tool for Audiovisual Materials (PEMAT-AV) was used to assess understandability (PEMAT-V) and actionability (PEMAT-A), each scored 0 to 100 [18]. The Journal of American Medical Association (JAMA) benchmark criteria was used to assess overall quality based on authorship, attribution, disclosure, and currency of the information provided in each video, with a maximum score of 4 [19]

### Statistical analysis

Categorical variables were summarized as frequencies and percentages; continuous variables were reported as medians with interquartile ranges (IQRs). Spearman’s rank correlation was used to assess associations between video engagement (views, VPI) and quality measures (PEMAT, JAMA benchmark). We also examined correlations between upload date and engagement metrics. Kruskal-Wallis test was used to examine the association between author type (academic, patient, or layperson) and video engagement/quality. Statistical significance was set to alpha < 0.05. All statistical analyses were performed in R Studio (version 4.2.3; Posit, PBC).

## Results

### Bladder exstrophy video characteristics

**Fig. 1:**
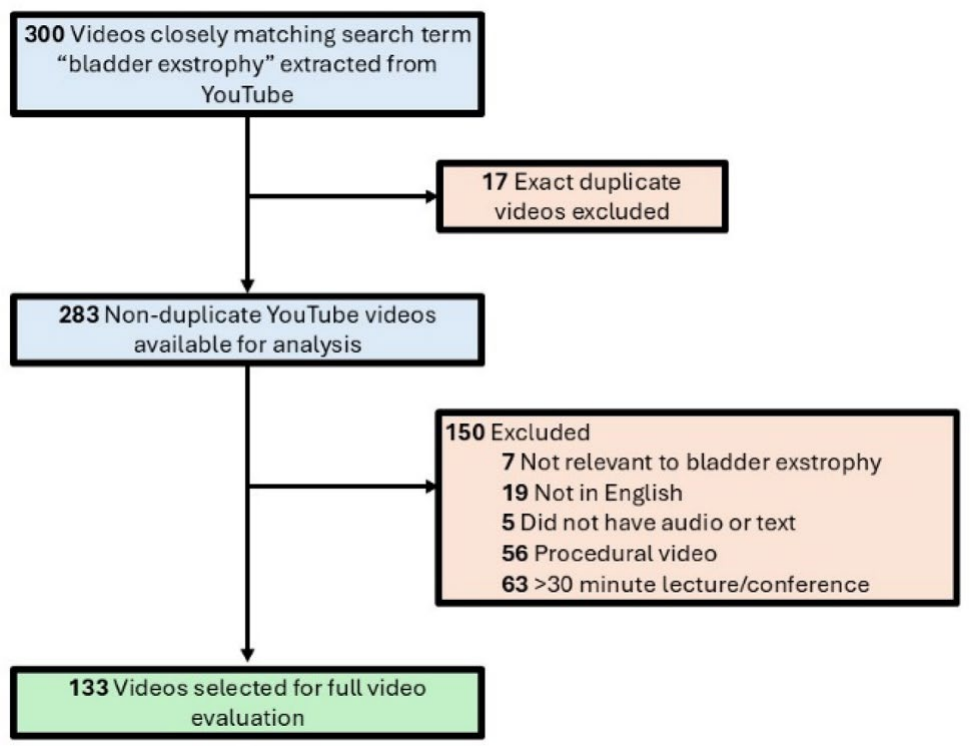
Flowchart of included YouTube videos selected from the top 300 videos appearing under search term “bladder exstrophy”

Of the 300 videos screened, 17 were duplicates. Among the 283 unique videos, 150 videos did not meet inclusion criteria, leaving 133 videos for full evaluation (Fig. 1).

Table 1 Summarizes video characteristics. Most videos (60%, *n* = 80) were produced by academic institutions with bladder exstrophy programs. One program was responsible for a quarter (*n* = 33) of all videos analyzed. Patient-created content accounted for 32, including two of the top three most prolific channels. The median video length was five minutes (IQR: 2.6, 6.9) and the median video age was 2.2 years (IQR: 1.9, 6.7). All videos included english text or audio, with the majority originating from North America (*n* = 96), followed by Australia/New Zealand (*n* = 15)

The median number of views was 204 (IQR: 43, 1755) and the median number of likes was 3 (IQR: 0,14). Video age correlated positively with view count (rho: 0.30, *p* < 0.001). However, there was not a significant association between video age and number of likes (rho:0.10, *p* = 0.26).

### Engagement in bladder exstrophy YouTube videos

Engagement and quality metrics are reported in Table 1. The median video power index (VPI) was 0.75 (IQR: 0,2.12). Older videos had significantly lower VPI (rho:−0.22, *p* = 0.013). By author type, patient-created videos had the highest median VPI (Median (IQR): 1.4 (0.30,5.52). However, this was not significantly different from academic (Median (IQR): 0.72 (0, 1.76)) and layperson (Median (IQR): 0.52 (0.03, 2.49) content producers (Supplement 2 A, *p* = 0.11). VPI did not vary by intended audience (Supplement 2B, *p* = 0.38). Regionally, videos from Australia had significantly higher VPI than those from North America (Supplement 2 C, *p* < 0.001). No other regional comparisons reached statistical significance. The majority of videos had no comments (*n* = 71) or disabled comments (*n* = 32). Of the 30 videos with comments, 83% were positive.

**Table 1:** Video metadata characteristics of YouTube videos on bladder exstrophy meeting the selection criteria. Descriptive characteristics are represented as percentages for categorical variables and median, interquartile range for continuous variables

Video Metadata	Value^1^
Author Type	
Academic	80 (60)
Patient	32 (24)
Layperson	21 (16)
Intended Audience	
Patient	71 (53)
Healthcare	34 (26)
General Audience	28 (21)
Video Length (Minutes)	4.9 (2.6, 6.9)
Time Since Video Upload (Months)	24.6 (23.9, 80.7)
Views	204 (43, 1755)
Likes	3 (0,14)
Comment Response	
No comments	71 (53)
Comments disabled	32 (24)
Positive	25 (19)
Negative	2 (2)
Mixed	3 (2)
Video Origin	
North America	96 (72)
Europe	4 (3)
East Asia	2 (2)
South Asia	12 (9)
Middle East/Africa	4 (3)
Australia/NZ	15 (11)

^1^Categorical variables shown as n (%). Continuous variables represented as median (IQR)

**Table 2:** Video popularity, quality characteristics represented by video power index (VPI) and PEMAT-A/V, JAMA benchmark respectively. Descriptive characteristics are represented as percentages for categorical variables and median, interquartile range for continuous variables

Video Metadata	Value^1^
PEMAT-V	89 (77, 91)
PEMAT-A	0 (0,67)
JAMA Benchmark	2 (2,2)
Video Power Index (VPI)	0.75 (0, 2.12)
Product advertisement in video?	10 (7.5)

^1^Binary variable shown as n (%). Continuous variables represented as median (IQR)

### Quality of available YouTube videos

Video quality was defined by PEMAT-A/V and the JAMA benchmark. Median understandability (PEMAT-V) was 89 (IQR: 77,91), while median actionability (PEMAT-A) was 0 (IQR: 0,67). Understandability differed significantly by author type (Supplement 3 A, *p* = 0.003); patient-created videos (Median (IQR): 75 (70,89)) had lower PEMAT-V scores than academic videos (Median (IQR): 89 (80,91); *p* = 0.002) but had similar understandability to layperson videos (Median (IQR): 90 (67,100); *p* = 0.18). There was not a significant association between PEMAT-V and VPI (rho:−0.16, *p* = 0.076). A similar null association was observed when examining the association between PEMAT understandability and views (rho:0.12, *p* = 0.18).

Actionability also varied by author type (Supplement 3B, *p* = 0.005); with layperson videos (Median (IQR): 0 (0,0)) scoring lower than academic (Median (IQR): 33 (0,100); *p* = 0.007) or patient-produced videos (Median (IQR): 0 (0,67); *p* = 0.004). There was no significant difference in PEMAT-A scores between academic and patient-authored videos (*p* = 0.52). There was not a significant association between PEMAT actionability and VPI (rho:−0.11, *p* = 0.23) or views (rho:−0.064, *p* = 0.47).

The median JAMA benchmark score was 2 (IQR: 2,2). Only one video, from an academic institution, provided sources. Twenty-four videos (18%) included a conflict-of-interest disclosure. Academic institutions had significantly higher JAMA scores (Median (IQR): 2 (2,3)) compared to both layperson (Median (IQR): 1 (0.75,2)) and patient (Median (IQR): 1 (1.5,2)) content creators (Supplement 3 C, *p* < 0.001). JAMA benchmark scores were not significantly associated with VPI (rho:−0.11, *p* = 0.21) or view count (rho:0.15, *p* = 0.096).

## Discussion

Families affected by bladder exstrophy often seek accessible, high-quality educational resources to better understand the condition, and YouTube has emerged as a frequently used platform for this purpose. In this study, we present the first systematic assessment of bladder exstrophy (BE) content on YouTube, examining video volume, geographic origin, engagement metrics, and overall content quality.

Most BE videos originated from North America and Australia/New Zealand, despite the condition’s consistent global prevalence (1 in 30,000–50,000 live births). Although non-English videos were excluded from analysis, they accounted for only 6.3% of extracted content, further reflecting disparities in digital production and academic infrastructure rather than disease burden [20]. Similar trends have been observed in other surgically managed conditions; for instance, Zhang et al. found that YouTube videos for laparoscopic gastrectomy were largely from well-resourced countries [2, 21]. These imbalances raise concern about global inequity in access to high-quality health information. Addressing this will require intentional efforts to develop inclusive, culturally appropriate, and multilingual educational materials, particularly for the low- and middle-income regions that remain underrepresented.

Engagement metrics in our study were modest, with a median of 204 views and 3 likes per video. As expected, older videos accrued more views over time, but they did not receive more likes, suggesting content longevity may improve visibility but does not increase user endorsement or interaction [16, 22]. Although YouTube holds promise as a platform for disseminating rare disease information, its current role in BE education appears limited and underutilized. Importantly, the overall quality analysis revealed strengths and weaknesses. Understandability (measured by PEMAT-V) was high, indicating effective communication of medical concepts. In contrast, the actionability (PEMAT-A) was remarkably low, with the majority of videos focused on patient experiences and pathophysiology rather than concrete steps in treatment or surgical timing. While it is appropriate that individualized care decisions be left to urologists following a thorough evaluation, families navigating a new diagnosis may benefit from prompts such as key questions to ask at consultation or frameworks outlining next steps in care. JAMA benchmark scores were also low, highlighting gaps in transparency regarding authorship, conflicts of interest, and source citation. These findings mirror trends observed in video content for other urologic conditions, with materials emphasizing explanation over actionable support [23]. Together, these results underscore the need for BE content that is not only informative but actionable, transparent, and better aligned with the practical needs of patients and families.

Our analysis suggests that video characteristics such as author type, region of origin, and age influence engagement patterns, although no single factor consistently predicts higher user interaction. Although not statistically significant, patient‑created videos generally showed greater engagement than those produced by academic or lay contributors, reflecting the potential resonance of lived experience. Videos from Australia appeared to receive higher engagement than those from North America, pointing to the role of regional production styles or even algorithm preferences in shaping visibility, independent of content quality. The trend of regional variations influencing engagement patterns regardless of caliber aligns with findings from studies on colorectal cancer and varioceles [21, 24]. Furthermore, the lack of active comment sections on most videos limits opportunities for engagement, peer support, and clarification; an important consideration for future content creators.

Despite clear differences in content quality across author types, with academic videos demonstrating greater clarity and transparency, these higher-quality materials did not generate greater engagement. The disconnect between educational value and viewer interaction is not unique to BE; similar trends have been observed across a range of medical conditions, where evidence-based content often struggles to attract visibility or emotionally connect with audiences [14, 21, 22, 24]. Platform algorithms that prioritize watch time, relatability, or emotionally-driven narratives may inadvertently suppress academically rigorous content, particularly when such videos lack humanizing elements. The predominance of positive but infrequent comments highlights this tension, with many academic channels disabling comment sections and thereby limiting meaningful dialogue. These findings suggest that improving the reach and impact of BE videos may require a tailored strategy to balance clinical accuracy with storytelling, emotional resonance, and practical guidance to meet real-world needs of patients and families.

### Limitations

Our study has several limitations. We focused exclusively on YouTube, excluding other widely used platforms such as TikTok and Instagram, which may be more popular among younger audiences and increasingly serve as sources of health information. As such, our findings may not capture all BE-related content, however, we intentionally selected a single platform to minimize bias in rating introduced by differed user interfaces and engagement norms to allow for a more consistent comparative analysis. As mentioned, the distribution of videos was heavily skewed toward North America and Australia/New Zealand, raising discussion about the potential disparities in online health education access. Additionally, while only 6.3% of the 300 screened videos were not in English and were excluded from full analysis, they may have offered unique perspectives or culturally relevant content that was not captured in our review.

While we used validated tools to assess video quality (PEMAT-A/V, JAMA benchmark), these have not been employed for examination of BE content. These measures also do not account for emotional resonance of content, which may explain the lower formal quality scores achieving higher engagement in patient-created content. Furthermore, the cross-sectional nature of our study limits our ability to examine how content and engagement evolve over time. All scoring was conducted by two individual reviewers (AS, AW) rather than average across multiple raters, which may introduce subjectivity despite standardized criteria [24]. 

Future research may benefit from improved regional representation, multi-platform assessments, partnerships with patients/families that incorporates qualitative user perspectives.

## Conclusion

This study represents the first systematic evaluation of bladder exstrophy content on YouTube, revealing that while videos are generally understandable (PEMAT-V), they often lack actionable guidance (PEMAT-A) and transparency (JAMA benchmark). Despite academic institutions producing the majority of content and achieving higher formal quality scores, these videos did not generate greater engagement, suggesting credibility alone does not drive visibility or user interaction. The predominance of content from high-resource regions and exclusion of non-English videos (6.3%) further highlight existing disparities in access to online health education. Patient-created videos, though lower in formal quality metrics, resonated more strongly with viewers, emphasizing the value of experiential narratives. These findings underscore a critical gap in educational resources for families affected by bladder exstrophy. To enhance the impact of future content, there should be multidisciplinary engagement to create inclusive, evidence-based materials that are practically useful. Addressing this need will be key to improve digital health equity for rare urologic conditions like bladder exstrophy.

## Supplementary Information

Below is the link to the electronic supplementary material.

Supplementary Material 1 Supplement 1: Custom Python script query to anonymously extract the top 300 videos matching the search team “bladder exstrophy”. Video title, publication dates, channel names, view count, like count, and URL were autonomously extracted through this method. Data was collected across pages using the API token and saved to a structured CSV file for analysis

Supplementary Material 2 Supplement 2: Video Power Index (VPI) measurement stratified by author type (A), targeted audience (B), and video origin (C), analyzed by KruskalWallis Rank Sum test. ‘***’ denotes p<0.001 statistical difference for higher VPI of videos of Australian/NZ origin compared to all other regions of video origin

Supplementary Material 3 Supplement 3: Video understandability (A), actionability (B), and JAMA benchmark (C) stratified by author type. ‘**’ denotes significantly lower video understandability for patient-produced videos compared to academic videos and significantly lower video actionability for layperson videos compared to patient or academic-produced videos. ‘***’ denotes p<0.001 statistical difference for higher JAMA benchmark of academic videos compared to patient or layperson-produced videos

## Data Availability

Data is available upon reasonable request.
