# Supplementary figures and images for: Bladder exstrophy on YouTube: an evaluation of quality, actionability, and user engagement
Source: World J Urol. 2026 Apr 11;44(1):297. doi: 10.1007/s00345-026-06229-z (PMC13070057; doi:10.1007/s00345-026-06229-z)

**A**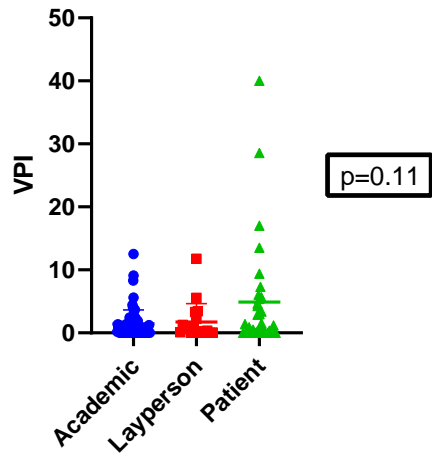**B**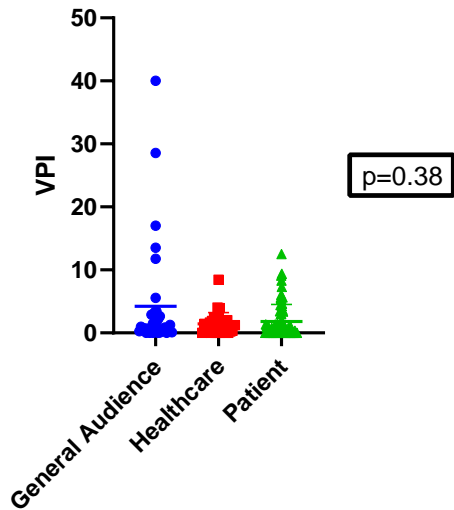**C**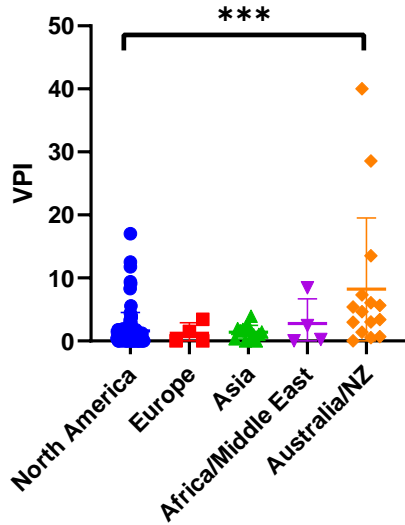

Supplement: Supplementary file 1 — Supplementary Material 1 Supplement 1: Custom Python script query to anonymously extract the top 300 videos matching the search team “bladder exstrophy”. Video title, publication dates, channel names, view count, like count, and URL were autonomously extracted through this method. Data was collected across pages using the API token and saved to a structured CSV file for analysis [file 345_2026_6229_MOESM1_ESM.pdf]

**A**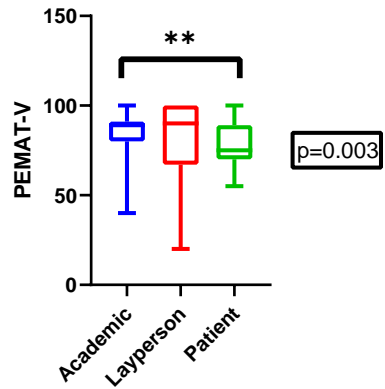**B**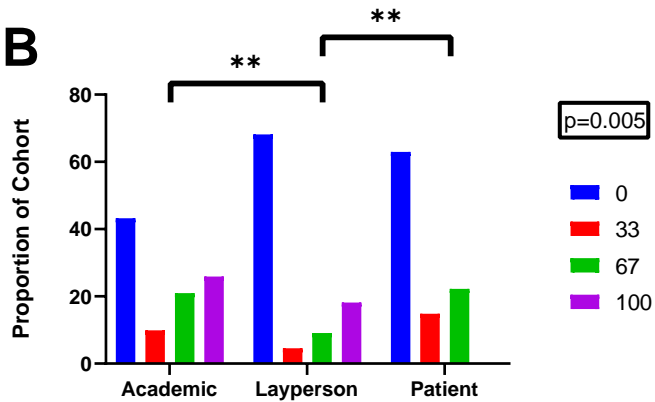**C**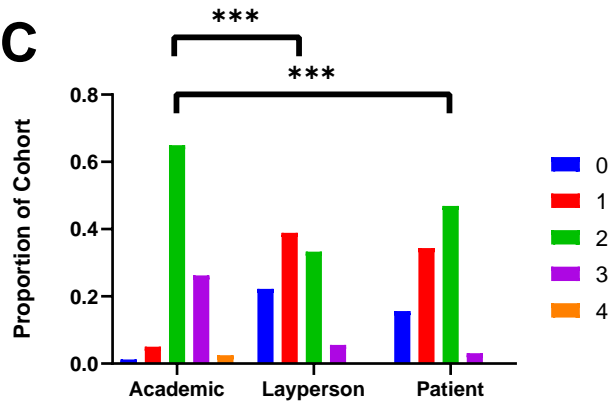

Supplement: Supplementary file 3 — Supplementary Material 3 Supplement 3: Video understandability (A), actionability (B), and JAMA benchmark (C) stratified by author type. ‘**’ denotes significantly lower video understandability for patient-produced videos compared to academic videos and significantly lower video actionability for layperson videos compared to patient or academic-produced videos. ‘***’ denotes p<0.001 statistical difference for higher JAMA benchmark of academic videos compared to patient or layperson-produced videos [file 345_2026_6229_MOESM3_ESM.pdf]
