# Supplementary material for: Bladder exstrophy on YouTube: an evaluation of quality, actionability, and user engagement
Source: World J Urol. 2026 Apr 11;44(1):297. doi: 10.1007/s00345-026-06229-z (PMC13070057; doi:10.1007/s00345-026-06229-z)
Supplement: Supplementary file 2 — Supplementary Material 2 Supplement 2: Video Power Index (VPI) measurement stratified by author type (A), targeted audience (B), and video origin (C), analyzed by KruskalWallis Rank Sum test. ‘***’ denotes p<0.001 statistical difference for higher VPI of videos of Australian/NZ origin compared to all other regions of video origin [file 345_2026_6229_MOESM2_ESM.docx]

import requests

import pandas as pd

import time

# 🔹 Replace with your YouTube API Key

API_KEY = "AIzaSyDqBM_WxQpLjObKYFDvG36oqNY1i67gjQo"

SEARCH_QUERY = "bladder exstrophy"

MAX_RESULTS = 300 # Number of videos to fetch

VIDEOS_PER_PAGE = 50 # YouTube API allows max 50 per request

video_data = []

# 🔹 Function to fetch videos

def fetch_videos(api_key, query, max_results):

url = "https://www.googleapis.com/youtube/v3/search"

params = {

"part": "snippet",

"q": query,

"type": "video",

"maxResults": VIDEOS_PER_PAGE,

"key": api_key

}

next_page_token = None

while len(video_data) < max_results:

if next_page_token:

params["pageToken"] = next_page_token

response = requests.get(url, params=params).json()

if "items" not in response:

print("Error:", response)

break

video_ids = [item["id"]["videoId"] for item in response["items"]]

fetch_video_stats(video_ids, response["items"])

next_page_token = response.get("nextPageToken")

if not next_page_token:

break # Stop if no more pages

time.sleep(1) # Avoid hitting API rate limits

# 🔹 Function to fetch video statistics

def fetch_video_stats(video_ids, video_items):

url = "https://www.googleapis.com/youtube/v3/videos"

params = {

"part": "statistics,snippet",

"id": ",".join(video_ids),

"key": API_KEY

}

response = requests.get(url, params=params).json()

for item in response.get("items", []):

snippet = item["snippet"]

stats = item.get("statistics", {})

video_info = {

"Title": snippet["title"],

"Video ID": item["id"],

"Channel": snippet["channelTitle"],

"Published Date": snippet["publishedAt"],

"Views": int(stats.get("viewCount", 0)),

"Likes": int(stats.get("likeCount", 0)),

"Video Link": f"https://www.youtube.com/watch?v={item['id']}"

}

video_data.append(video_info)

# 🔹 Fetch data

fetch_videos(API_KEY, SEARCH_QUERY, MAX_RESULTS)

# 🔹 Save to DataFrame

df = pd.DataFrame(video_data)

# 🔹 Save to CSV

csv_filename = "bladder_exstrophy_videos.csv"

df.to_csv(csv_filename, index=False)

# 🔹 Display completion message

print(f"✅ Data extraction complete! CSV saved as: {csv_filename}")
